# A case of Bietti crystalline dystrophy with preserved visual acuity and extinguished electroretinogram: a case report

**DOI:** 10.4076/1757-1626-2-7100

**Published:** 2009-08-12

**Authors:** Ali Tabatabaei, Mohammad Soleimani, Sasan Moghimi, Mohammad Yaser Kiarudi

**Affiliations:** Department of Ophthalmology, Farabi Eye Research CenterSouth Kargar Street, Tehran, Postal code: 1336616351Iran

## Abstract

**Introduction:**

Progressive night blindness and constriction of the visual fields are features of Bietti crystalline corneoretinal dystrophy, but here we report a case with the most probable diagnosis of Bietti crystalline dystrophy and good central visual acuity and severely decreased electroretinogram.

**Case presentation:**

The patient was a 28-year-old woman with complaint of decreased vision without night blindness. Her both eyes visual acuity were 20/25 with plano refraction. Fundus examination showed intraretinal crystals distributed in the posterior pole and also midperiphery. Fullfield electroretinogram showed decreased scotopic a and b-wave amplitudes.

**Conclusion:**

In our patient central foveal region was relatively intact; and this can explain subnormal visual acuity. Although visual acuity was nearly spared, electroretinogram was extremely affected.

## Introduction

Bietti crystalline corneoretinal dystrophy (BCD) is an autosomal recessive disorder of retinal degeneration characterized by numerous tiny sparkling yellow-white spots at the posterior pole of the fundus. The small glistering crystals can also occur in the corneal limbus and circulating lymphocytes [1,2]. Clinically, affected patients experience decreased vision, night blindness, and constriction of the visual fields, usually around the third and forth decade of life [1,2]. BCD is a rare retinal disorder worldwide, however it appears to be relatively common in China and Japan, where the gene frequency has been estimated to be 0.005 [3,4]. In this article, we reported a case with the most probable diagnosis of Bietti crystalline dystrophy (BCD). To our knowledge, this is the first case report of this denegation in Iran.

## Case presentation

A 28-year-old Iranian Caucasian woman referred to our clinic with complaints of decreased vision without night blindness and visual acuity of both eyes was 20/25 with plano refraction. Her slit lamp examination revealed clear cornea and clear lens, and 1+ pigment in vitreous. Intraocular pressure was 18 mmHg for right eye and 17 mmHg for left eye. She did not have any history of associated other medical conditions or drug usage.

**Figure 1.**
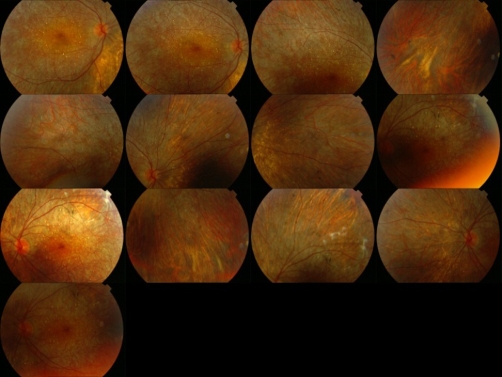
Fundus photograph of Bietti crystalline corneoretinal dystrophy (BCD).

Fundus examination showed intraretinal crystals distributed in the posterior pole and also midperiphery. There was midperipheral RPE and choriocapillaris atrophy, peripheral pigment clumping and retinal scarring (Figure 1). Flourescein angiography highlighted the focal geographic appearance showing transmission hyperfluorescence in the crystalline retina and choriocapillaris atrophy in the adjacent noncrystalline retina.

**Figure 2.**
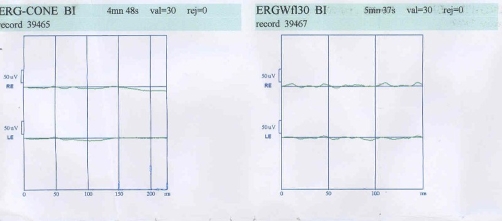
30-Hz flicker response and cone response ERG of both eyes showing severely decreased both a and b waves.

**Figure 3.**
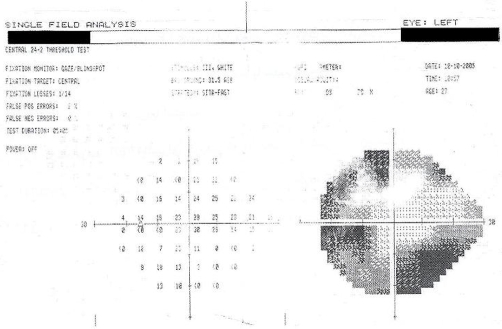
Visual field testing with program SITA fast 24 -2 showed constriction of visual field.

Fullfield ERG showed severely decreased scotopic a and b-wave amplitudes. Moreover, the photopic a and b wave amplitudes were both severely attenuated (Figure 2). Visual field testing with program SITA fast 24 -2 was performed and showed constriction of visual field bilaterally (Figure 3).

## Conclusion

Bietti first reported cases of tapetoretinal degeneration characterized by yellow glistering retinal crystals, choroidal sclerosis, and marginal crystalline dystrophy of the cornea. Similar cases with no limbal crystalline deposits have been reported and called Bietti crystalline fundus dystrophy or crystalline retinopathy [5-8]. The patient in the present study also had no corneolimbal crystals.

Affected patients presented with visual symptoms from the third decade onward. The most common presenting symptom is a decrease in visual acuity, often accompanied by nyctalopia. In the presented patient, visual acuity was good and central vision had not been affected. This sparing of the central vision until late in the disease is also recently described in European patients with BCD [9].

Patient with RPE atrophy and intraretinal crystals confined to the posterior pole tend to show lesser disturbances in the full-field electroretinogram (ERG) [10]. This is in contrast to Stargardt’s disease, in which the distribution of flecks is not a good indicator of the extent of retinal dysfunction, as demonstrable on full-field ERG [11].

Histologically, BCD shows evidence of advanced panchorioretinal atrophy characterized by generalized loss and sclerosis of the choriocapillaris with crystals and complex lipid inclusions within the choroidal fibroblasts. The abnormal inclusions are similar to those found in circulating lymphocytes, keratocytes, and conjunctival and skin fibroblasts [1,2]. According to these data, BCD may belong to the systemic abnormalities of lipid metabolism. Cultured lymphocytes from BCD patients lack two fatty acid-binding proteins of 32 and 45 kDa, in comparison to age-matched controls [12].

Bietti initially noted similarities with retinitis punctata albescens and fundus albipunctatus. These conditions are considered flecked retina syndromes and do not have the distinctive crystalline deposits in patients with Bietti’s crystalline dystrophy. Several advanced cases of Bietti’s crystalline dystrophy have been misdiagnosed as retinitis pigmentosa, since advanced cases may have severe visual loss, extensive chorioretinal atrophy, pigment deposition, minimal crystals, and nonrecordable ERG. The diagnosis of BCD is based on clinical findings; biomicroscopic and ophthalmoscopic appearance are usually sufficient to make diagnosis [13]. We can confirm the diagnosis with genomic study or fibroblast and lymphocyte cultures or electron microscopy trial [10]. Our patient refused to do genetic examination or biopsy of retina for electron microscopy trial or blood sampling. In fact, we can consider other diffrential diagnosis such as retinitis punctata albescens, fundus albipunctatus,retinitis pigmentosa, Stargardt’s disease, autosomal dominant crystaline dystrophy, etc.

## Abbreviations

BCD: Bietti crystalline corneoretinal dystrophy
ERG: electroretinogram
RPE: retinal pigment epithelium
SITA: Swedish interactive thresholding algorithm
